# Surgical anatomy of the internal thoracic lymph nodes in fresh human cadavers: basis for sentinel node biopsy

**DOI:** 10.1186/s12957-016-0897-2

**Published:** 2016-04-30

**Authors:** Alfredo Carlos S. D. Barros, Lincon Jo Mori, Dolores Nishimura, Alfredo L. Jacomo

**Affiliations:** Discipline of Human Structural Topography, University of São Paulo Medical School, Av. Dr. Arnaldo, 455, São Paulo, SP 01246-903 Brazil; Mastology Department, Sírio-Libanês Hospital, Rua Adma Jafet, 91, São Paulo, SP 01308-000 Brazil; Rua Dr. Renato Paes de Barros, 750 cj 35, São Paulo, SP 04530-001 Brazil

**Keywords:** Internal thoracic lymph nodes, Sentinel node biopsy, Breast cancer

## Abstract

**Background:**

While the optimal management of early breast cancer patients with sentinel lymph node (SLN) involvement mapped in the internal thoracic chain is still debated, biopsy may be performed when surgeons select patients who are most likely to benefit.

The aim of this study is to examine anatomical aspects of internal thoracic nodes (ITNs) to orientate SLN biopsy in the parasternal area.

**Methods:**

This study was based on dissections of 29 female cadavers. The parameters analyzed were the number of intercostal spaces (ICSs) containing at least one ITN, mean number of nodes in each ICS, position of the ITNs in relation to the internal thoracic artery (ITA), number of retrocostal spaces (RCSs) containing at least one ITN, and mean number of nodes in each RCS.

**Results:**

The ICS that was most likely to have at least one ITN was the third, with 86.2 % in the right side and 75.8 % in the left side. In the second ICS, the rates were 69.2 and 73.6 %, and in the fourth, the rates were 48.1 and 33.3 %. In the third ICS, on both sides, the mean number of ITNs was the highest (1.2). A tendency of the nodes to be laterally located in the second ICS and medially located in the downward dissection was observed. Most of the RCSs did not present any nodes.

**Conclusions:**

This study indicates that most of the second and third ICSs presented at least one ITN, and the mean number of nodes in the third space was greater. There is a tendency to find nodes medial to the artery downwards from the second to the fourth ICS. ITNs are generally located in ICSs, and the majority of RCSs did not contain any nodes.

## Background

Lymphatic drainage of the breast is of great importance in the process of breast cancer (BC) metastastization. After absorbing lymph from the interstitial space, occasionally carrying tumor cells, lymphatic capillaries drain unidirectionally into the collecting lymphatic vessels, which in turn drain to lymph nodes (LNs). Efferent LN channels form large trunks that discharge into the venous circulation [1]. Intramammary lymphatics flow toward axillary and/or internal thoracic nodes (ITNs) [1, 2]. Although the axillary nodal basis is the most common dissemination pathway, the status of ITNs shares similar prognostic relevance as reflected in the AJCC staging system [3].

The sentinel lymph node (SLN) is the first node that drains a cancer [4]. If this node is clean of metastasis in early BC, no further axillary nodal dissection is indicated [5]. Axillary SLN biopsy is a procedure that is used for the staging and therapeutic guidance of patients with clinically node-negative early infiltrating BC [6].

Lymphoscintigraphic studies in BC patients have shown a significant proportion of cases with drainage to ITNs, including approximately 30 % of the medial tumors and 15 % of the lateral tumors [7]. Nevertheless, to date the value of SLN biopsy when located in the internal thoracic chain (ITC) is controversial, as it seems not to influence survival outcomes for most patients [7–9]. However, Madsen et al. showed that the small subgroup of patients who had ITNs metastases without axillary involvement had worse outcome than patients without any regional lymph node metastases [10]. For some authors, the assessment of SLN in the ITC should be considered if it is feasible and informative, leading to more accurate staging and potential changes in adjuvant radiotherapy and/or chemotherapy [11–14].

It is out of the scope of our research to elucidate this controversy. We aim to examine the topographic anatomical aspects of ITNs in fresh human cadavers to orientate SLN biopsy in ITC when surgeons select patients who are most likely to benefit. The main points addressed were the presence of ITNs in the second, third, or fourth intercostal spaces (ICSs) as well as in the retrocostal spaces (RCSs) of the chest wall and their positioning in relation to the internal thoracic artery (ITA), which is the landmark for safe dissections in the parasternal region [15].

## Methods

This was a prospective study based on anatomical dissections of 29 fresh adult female human cadavers performed at a single institution, University of São Paulo Medical School, in the Discipline of Human Structural Topography. All of the deaths occurred up to 12 h before the dissections. The ages of the deaths varied between 22 and 81 years (median 57.4). Prior to commencing the study, the research protocol obtained approval from our Institutional Ethical Committee.

### Dissection technique

**Fig. 1:**
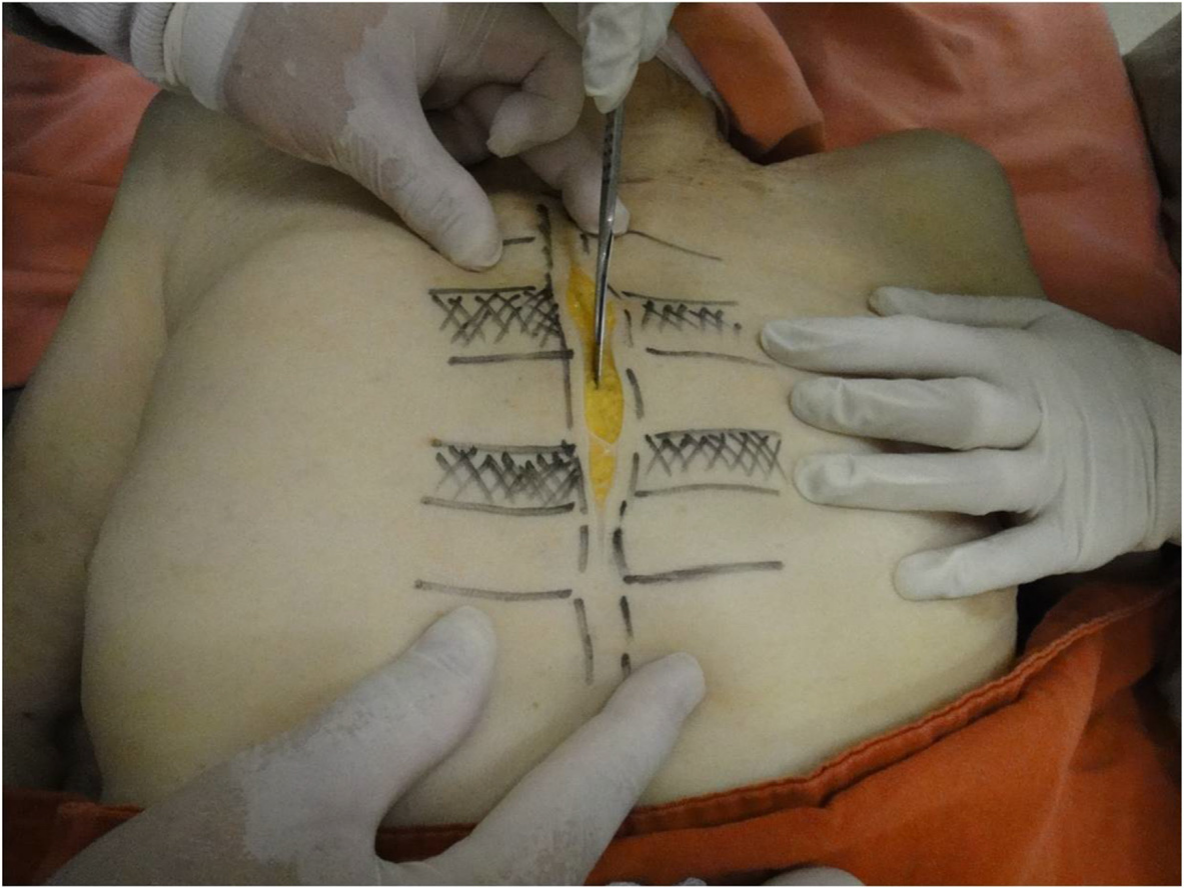
Skin incision over the sternum

**Fig. 2:**
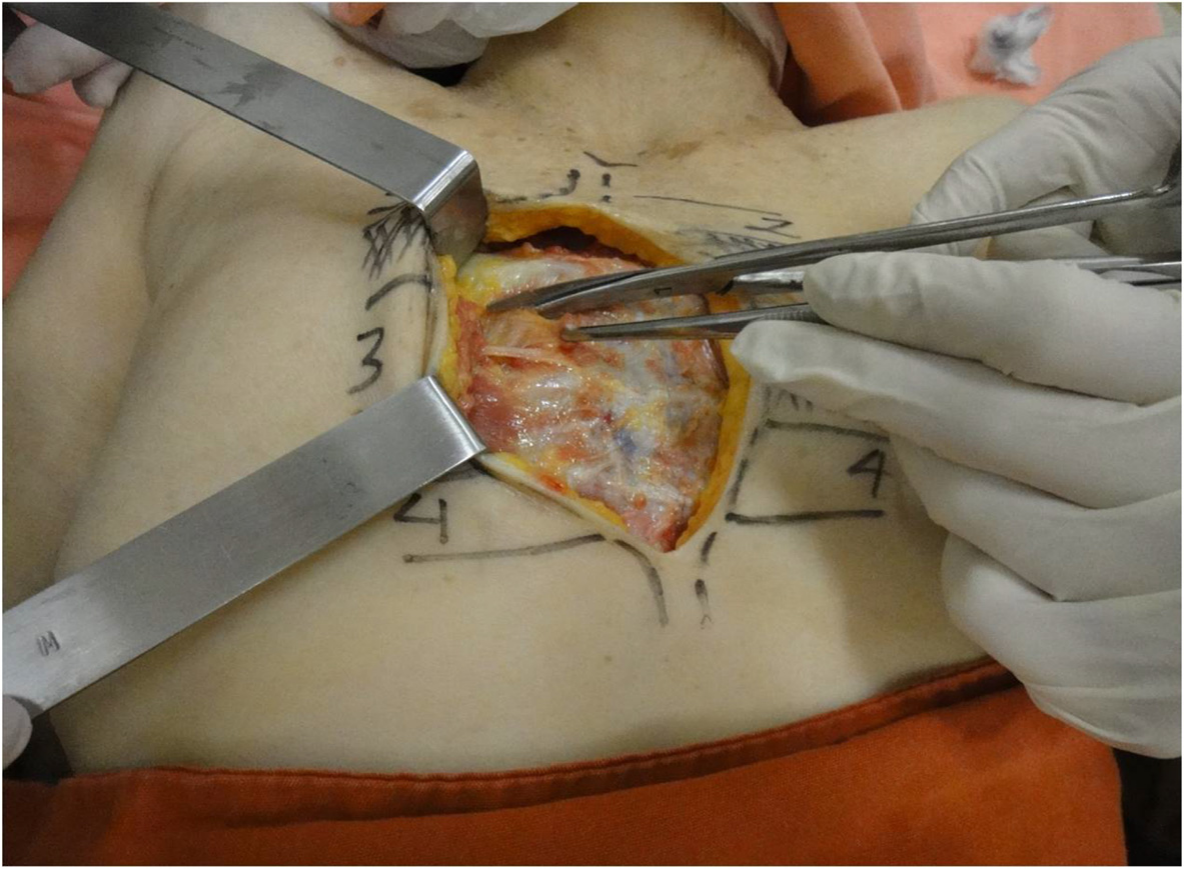
Section of the intercostal muscles

**Fig. 3:**
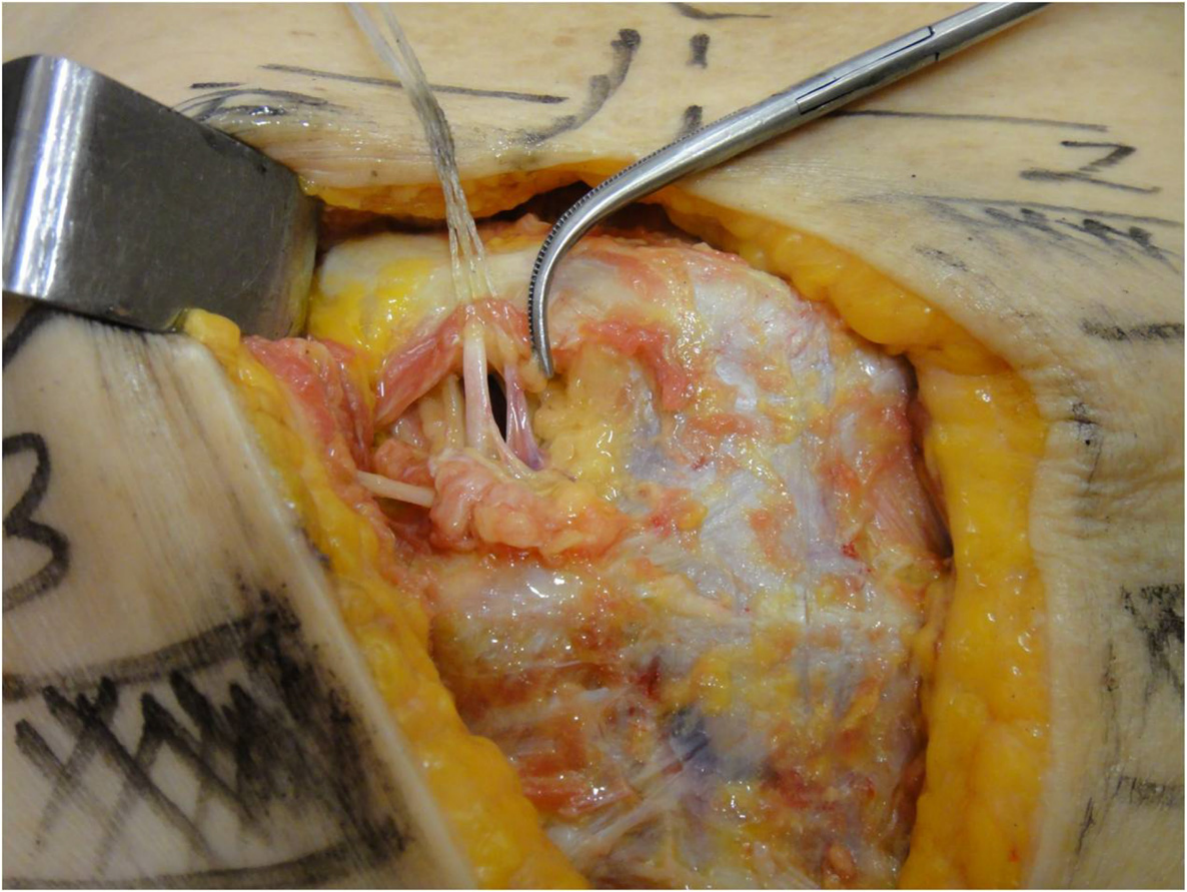
Excision of an internal thoracic lymph node medially located in relation to the repaired artery and vein

**Fig. 4:**
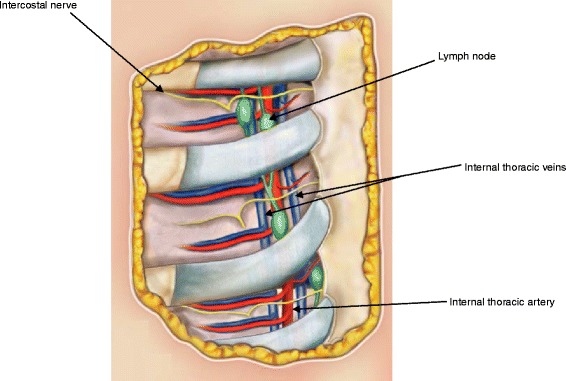
Anatomical relationships of the internal thoracic lymph nodes in the intercostal spaces

**Fig. 5:**
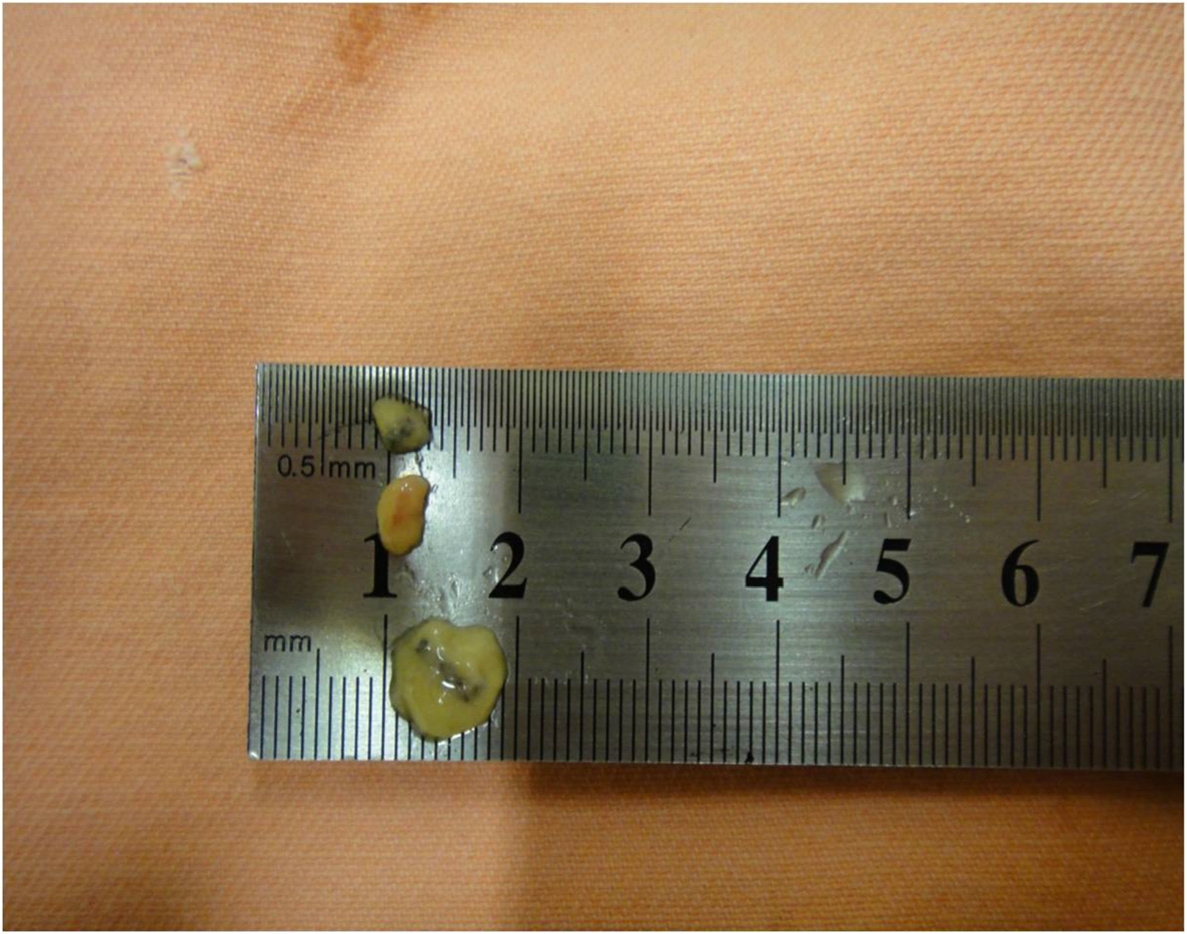
Excised internal lymphatic lymph node

**Fig. 6:**
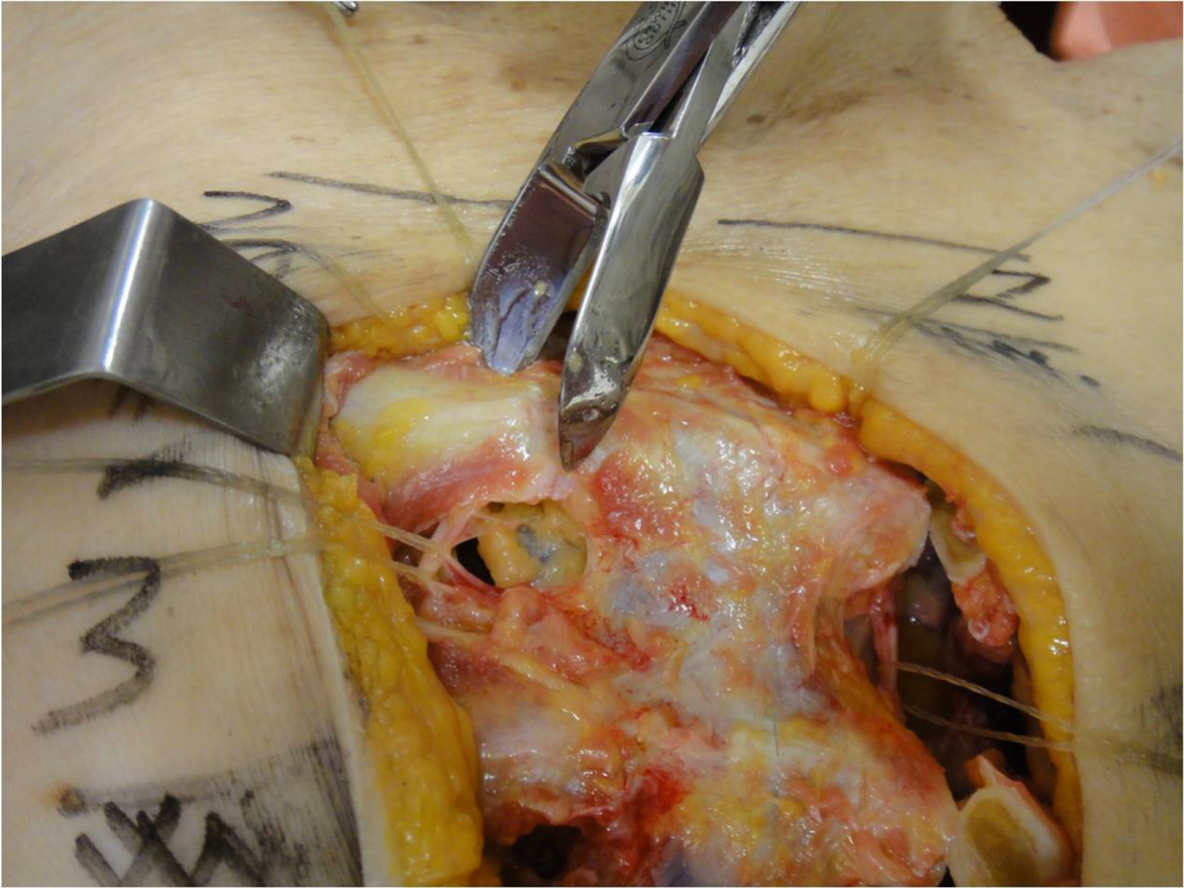
Costal cartilage cut in its sternal junction

**Fig. 7:**
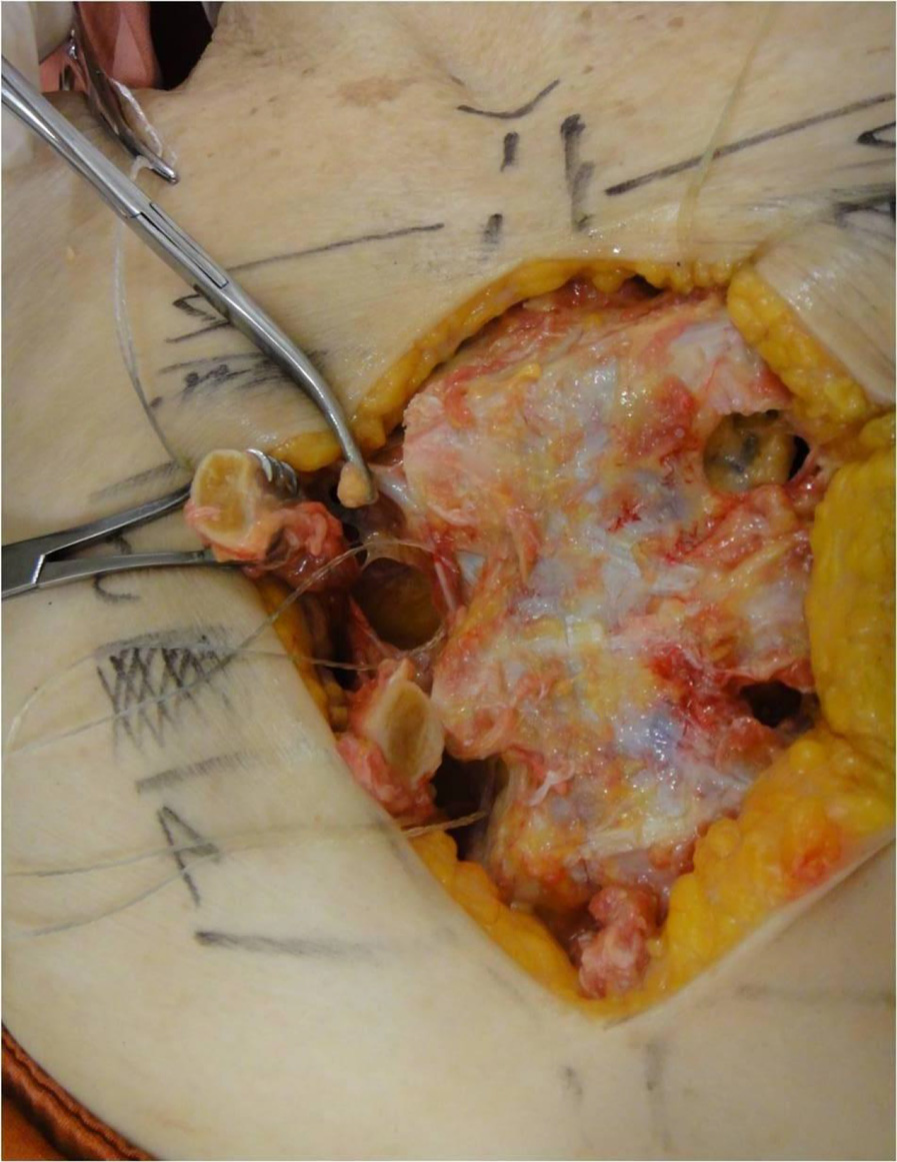
Retrocostal lymph node harvested after rib section and traction

The dissection followed a step-by-step standardized protocol:

1. Median longitudinal incision in the overlying sternum skin (Fig. 1)
2. Pectoralis major muscle desinsertion from the lateral sternal border at both sides of the thorax followed by its lateral traction
3. Bilateral exposition of the second, third, and fourth ICSs
4. Section of the intercostal muscles (Fig. 2) and careful opening of the thin anterior leaflet of the parietal pleura in the parasternal area
5. Cautious scissor dissection to expose and repair the ITA just over the posterior layer of the parietal pleura
6. Identification of the ITNs surrounded by fatty tissue and observation of their positioning in relation to the ITA (Figs. 3 and 4)
7. Excision of any LNs found, which are recognized as hard small corpuscular structures, measuring 1.0–10.0 mm (Fig. 5)
8. Cut the costal cartilages in their sternal junctions using a small costotome (this procedure was performed, as shown in Fig. 6, for 19 out of the 29 cadavers)
9. Ribs traction toward the lateral side of the chest to allow RCS dissections (Fig. 7)
10. Wound closure

### Data analysis

This was a descriptive study with an analysis of the frequency of the findings. The following parameters were analyzed: the number of ICSs containing at least one LN, mean number of LNs in each ICS, the position of the LNs in relation to the ITA, number of RCSs containing at least one LN, and mean number of LNs in each RCS.

## Results

### ITLN presence in ICSs

The number of ICSs containing at least one LN is presented in Table 1. The ICS that was more likely to have at least one LN was the third, with 25/29 in the right side (86.2 %) and 22/29 in the left side (75.8 %). Thus, the likelihood of having LNs in the second and fourth ICS was lower. It is particularly noteworthy that more than half of the fourth ICSs did not reveal any LN.

**Table 1:** Number of intercostal spaces containing at least one internal thoracic lymph node

	Hemithorax	
	Right *n* (%)	Left *n* (%)
Second (26)^a^	18 (69.2)	19 (73.6)
Third (29)	25 (86.2)	22 (75.8)
Fourth (27)^b^	13 (48.1)	9 (33.3)

^a^Three occluded spaces

^b^Two occluded spaces

Table 2 lists the mean number of retrieved LNs in each ICS, calculated by the relationship between the total number of LNs found in a specific space and the number of the corresponding spaces dissected. In the third, at both sides, the mean number was the highest (1.2; range 0–4).

**Table 2:** Mean number of lymph nodes found in each of the intercostal spaces

	Hemithorax	
Intercostal space	Right	Left
Second (26)^a^	0.8 (23/26)	1.1 (29/26)
Third (29)	1.2 (37/29)	1.2 (36/29)
Fourth (27)^b^	0.7 (19/27)	0.7 (19/27)

^a^Three occluded spaces

^b^Two occluded spaces

### Position of the ITLNs in the ICSs in relation to the internal thoracic artery

The relationship between the ITNs and the ITA was not uniform, but at both sides, there was a tendency of the LNs to be laterally located in relation to the artery in the second spaces and medially in the downward direction. It was possible to observe that in the third and fourth spaces, the LNs were mainly located at the medial side of the artery (Tables 3 and 4).

**Table 3:** Position of the internal thoracic lymph nodes in relation to the internal thoracic artery in the different intercostal spaces in the right hemithorax

	Position			
Intercostal space	Medial *n* (%)	Lateral *n* (%)	Anterior *n* (%)	Posterior *n* (%)
Second (23 lymph nodes)	7 (30.4)	17 (52.1)	3 (13.0)	1 (4.3)
Third (37 lymph nodes)	19 (51.3)	16 (43.2)	1 (2.7)	1 (2.7)
Fourth (19 lymph nodes)	12 (63.1)	5 (26.3)	2 (10.5)	0 (-)

**Table 4:** Position of the internal thoracic lymph nodes in relation to the internal thoracic artery in the different intercostal spaces in the left hemithorax

	Position			
Intercostal space	Medial *n* (%)	Lateral *n* (%)	Anterior *n* (%)	Posterior *n* (%)
Second (29 lymph nodes)	11 (37.9)	16 (55.1)	1 (3.4)	1 (3.4)
Third (36 lymph nodes)	21 (58.3)	11 (30.5)	2 (5.5)	2 (5.5)
Fourth (19 lymph nodes)	11 (57.4)	6 (31.5)	3 (15.7)	1 (5.2)

### ITLN presence in retrocostal spaces

Most of the RCs did not present any LN. There were few internal thoracic lymph nodes (ITLNs) found in the second and third retrocostal regions, and they were rare behind the fourth costal cartilage (Tables 5 and 6).

**Table 5:** Number of retrocostal regions containing at least one internal thoracic lymph node

	Hemithorax	
Retrocostal space	Right *n* (%)	Left *n* (%)
Second (17)^a^	5 (29.4)	3 (17.6)
Third (19)	7 (36.8)	7 (36.8)
Fourth (19)	1 (5.2)	2 (10.5)

^a^Two not explored

**Table 6:** Mean number of lymph nodes found in each of the retrocostal regions

	Hemithorax	
Retrocostal space	Right *n* (%)	Left *n* (%)
Second (17)^a^	0.3 (5/17)	0.2 (3/17)
Third (19)	0.3 (7/19)	0.3 (7/19)
Fourth (19)	0.0 (1/19)	0.1 (2/19)

^a^Two not explored

## Discussion

Suami et al. studied fresh cadavers to examine the breast lymphatic drainage in detail [16]. According to their findings, lymphatic capillaries were found to be evenly spaced at the periphery of the anterior upper torso draining radially into the axillary LNs. As they reached the breast, some passed over and some passed through the parenchyma. They also observed perforating lymph vessels that coursed beside the branches of the internal thoracic vessels that drained into the ITC.

It is estimated that at least 97 % of the total lymph from the breast flows to axillary nodes, while only 3 % flows to ITNs [17]. Turner-Warwick described that there are basically three intercommunicating lymphatic plexuses involved in the drainage: superficial, perforating, and deep [1]. The superficial and perforating plexuses drain almost exclusively to the axillary nodes through the subareolar Sappey lymphatic network. The deep system drains to the axilla and to ITC [1, 2].

Deeply located malignant lesions have a greater chance to be drained by the deep plexus and consequently to spread via ITNs. However, as the intermediate perforating plexus is connected to the deep plexus, BC diagnosed in every part of the gland, in theory, has the potential to metastasize via ITNs. The prevalence of ITN drainage reflects the method of lymphoscintigraphy, where the peritumoral injections of radioisotopes (deep lymphatic plexus) have a much higher likelihood of ITN drainage than subdermal or subareolar injections (superficial lymphatic plexus).

We published elsewhere that a single injection of a colloidal solution labeled with ^99m^Technetium directly into the center of small non-palpable lesions under imaginologic guidance with the goal of simultaneous occult lesion localization and SLN mapping comprises a precise model to verify breast lymphatic pathways [18]. The first draining node was mapped only in the axilla in 86.6 % of the cases, only in the ITC in 4.5 % of the cases and concomitantly in the axilla and ITC in 8.9 % of the cases [19].

For Shimazu et al., if the tumor was situated in the medial part of the breast or deeply located in any part of the glandular tissue, the possibility of finding a SLN in the internal thoracic pathway was higher [20]. Estourgie et al. [15] mapped SLNs exclusively in the ITC in 5.8 % of the neoplasias located in the inferomedial quadrants and in 2.6, 1.5, and 1.1 % of the tumors in the superomedial, superolateral, and inferolateral quadrants, respectively.

ITN biopsy is safe when a skillful surgeon knows the local anatomy and operates with gentle sharp and blunt dissection. The ICSs are narrow and contain fine vessels, and the ITNs are confined between the two leaflets of the parietal pleura, 1.0–3.0 cm from the sternum (more laterally, the parietal pleura forms a single thicker membrane).

The ITA runs alongside the sternal border and is flanked by two parallel veins (one medial and another lateral), just next to the sternum in the first ICS, progressively increasing the distance from its margin to 1.5–2.0 cm in the downward direction. Two anterior and one posterior intercostal branches originate from the artery in each ICS. In the sixth space, the artery divides into two terminal branches, the abdominal and the musclefrenic. The internal thoracic veins join at the level of the first rib and discharge into the brachiocephalic trunk.

The ITLNs were found predominantly in the ICSs rather than in the RTCs behind the costal cartilages. We identified approximately five times more LNs in the ICSs, which makes the LN biopsy in this chain easier. This is why 80–93 % of the SLN in the ITC are excised with success without removing segments of the costal cartilages [21, 22].

When we retrieved one LN in an ICS, it was frequent to find another in the same space. For example, when the second ICSs had at least one LN, the mean number of LNs dissected in the same space was 1.4. Under the same conditions, the mean number of LNs was 1.5 in the third space and 1.7 in the fourth. The surgeon must bear in mind that the first node observed is not always the true SLN, justifying the radioguided biopsy under a gamma ray-detecting probe guidance. The detector is inserted into the spaces at different points to check the hottest spot.

Based on our cadaver dissections, surgeons must be prepared to find a SLN on either side of the ITA. Nevertheless, we observed that in more than half of the cases, the ITNs are lateral to the artery in the second ICSs, and, more commonly, medially situated in the third or fourth.

One of the main complications of the SLN in the ITC biopsy is bleeding caused by inadvertent injury to the internal thoracic vessels. The control of bleeding is performed by vessel ligation or clipping. When the vessel withdraws, the resection of a costal cartilage may be required to improve access. To prevent this complication, we recommend exposing and repairing the artery before starting the SLN harvest.

Simple opening of the pleural cavity without pneumothorax is another relatively common accident. There are two procedures for closing the defect, both of which are performed after lung hyperinsufflation: direct suture or application of a plug of absorbable hemostatic cellulose polymers. In more severe lesions, when a pneumothorax is formed, drainage becomes necessary.

Up to 25 % of BC patients have ITN infiltration, but at least two renowned studies established that complete dissection of the internal thoracic lymphatic pathway does not improve the outcome but rather increases the morbidity rate [23–25]. The routine of full ITC clearance has thus been abandoned. Even so, the introduction of the procedure of SLN biopsy in the ITC has renewed interest in the status of ITLNs, because it can modify adjuvant therapy for BC patients without causing significant rise in morbidity [26]. For example, according to Caudle et al. and Ozmen et al., SLN-ITC involvement altered adjuvancy, respectively, in 7 and 15.2 % of the patients in whom SLN biopsy was performed after preoperative lymphoscintigraphic showed drainage into the ITC [13, 14].

Although the ITC is, along with the axilla, a site of first and direct lymphatic drainage for BC, at this point in time, the optimal management of SLN in this lymphatic pathway is still debated [7, 27, 28]. According to Cody III and Sacchini, there are basically three conditions that justify SLN biopsy in ITC: (I) the SLN exclusively mapped in this chain; (II) the SLN mapped concomitantly in the axilla and in the ITC, the axillary SLN is benign on intraoperative examination, and the patient does not qualify as a candidate for adjuvant chemotherapy on the basis of other criteria; and (III) a second SLN biopsy after local cancer recurrence with SLN mapped in the ITC [29]. Precise knowledge of the topographic anatomy of the region containing the ITNs is paramount for performing successful SLN retrievals.

## Conclusions

In conclusion, we found that the topographic anatomy of the ITNs varies according to each woman. Still, it was observed that most of the second and third ICSs presented at least one LN and that the mean number of LNs in the third space was greater. There was a tendency to find LNs situated medially to the ITA downwards from the second to the fourth ICSs. SLNs in the ITC are generally located in the ICSs, and most of the retrocostal spaces did not contain any LNs.

## Abbreviations

ICS: intercostal space
ITA: internal thoracic artery
ITC: internal thoracic chain
ITN: internal thoracic node
LN: lymph node
RCS: retrocostal space
SLN: sentinel lymph node
